# Inter-observer and Intra-observer Reliability of Posterior Malleolus Fracture Classification Systems

**DOI:** 10.7759/cureus.95154

**Published:** 2025-10-22

**Authors:** Barry Mullins, Gregory Neal-Smith, Alisdair Felstead, John McFall, Harold Akehurst, Andrew Jowett, Togay Koç

**Affiliations:** 1 Trauma and Orthopaedics, Portsmouth Hospitals University National Health Service (NHS) Trust, Portsmouth, GBR; 2 Trauma and Orthopaedics, Salisbury National Health Service (NHS) Foundation Trust, Salisbury, GBR; 3 Trauma and Orthopaedics, Bristol Royal Hospital for Children, Bristol, GBR; 4 Trauma and Orthopaedics, University Hospital Southampton National Health Service (NHS) Foundation Trust, Southampton, GBR; 5 Bone and Joint Research Group, University of Southampton, Southampton, GBR

**Keywords:** ct, haraguchi classification, mason and molloy, posterior malleolus, posterior malleolus fracture, trauma and orthopedics

## Abstract

Introduction

The morphology of posterior malleolar fractures is recognized as an important variable in the management of ankle fractures. The classification systems for these fractures reflect morphological differences among them. In this study, we compared the inter-observer and intra-observer reliability of three classification systems for posterior malleolar fractures.

Methods

Forty computed tomography scans demonstrating ankle fractures with posterior malleolar components were reviewed by four reviewers on two separate occasions using the Mason and Molloy, Haraguchi, and Bartoníček classification systems. The reviewer group included two consultant foot and ankle surgeons, one foot and ankle fellow, and one specialist registrar. All members of the group were familiar with the three classification systems. We conducted a study of inter-observer and intra-observer reliability using the Fleiss kappa (κ) and mean Cohen’s kappa (κ) coefficients, respectively, using R software.

Results

The Fleiss kappa statistic for inter-observer reliability was 0.43 (95% CI 0.35-0.50) for the Bartoníček classification, 0.65 (0.5-0.75) for the Haraguchi classification, and 0.63 (0.55-0.72) for the Mason and Molloy classification. The mean Cohen’s kappa values for intra-observer reliability by classification were 0.66 (range 0.58-0.78), 0.73 (range 0.63-0.84), and 0.65 (range 0.61-0.70), respectively, for these classifications.

Conclusion

The Haraguchi classification had the highest inter-observer and intra-observer reliability. The inter-observer reliability agreement was substantial (0.61-0.80) for the Haraguchi classification and the Mason and Molloy classification. The Bartoníček classification demonstrated the lowest inter-observer reliability. Future research should assess the effect of these classification systems on decision-making and patient-reported outcomes.

## Introduction

The treatment of posterior malleolus fractures poses a significant challenge to trauma surgeons, not least because of the poor prognosis that is widely recognized to be associated with these injuries [1,2]. Historically, the proportion of articular involvement on standard radiographic imaging was judged to be the determining factor regarding whether the posterior malleolar component of a fracture should be addressed [3]. Recently, there has been increasing recognition of the importance of the posterior malleolus for tibiotalar and tibiofibular stability in the context of ankle fractures as well as evidence that the articular incongruency and impaction of the tibial plafond have a detrimental effect on patient outcomes [4,5].

A more recent opinion is that the morphology of a posterior malleolus fracture should determine the need for fixation [1]. Several classification systems have been created to assess the morphology of posterior malleolus fractures. Numerous research articles have demonstrated that plain radiography is neither sensitive nor specific enough to assess posterior malleolus injuries [6,7]. Therefore, each of these classification systems relies on computed tomography (CT) as the imaging modality.

Haraguchi et al. completed a CT-based study of the pathoanatomy of 57 posterior malleolar fractures [8]. This study had two aims: (i) to calculate the posterior fragment area relative to the cross-sectional area of the tibial plafond and (ii) to calculate the angle between the fracture line and the bimalleolar axis. From this analysis, three categories were created: posterolateral-oblique (type 1), medial-extension (type 2), and small-shell (type 3) [8]. Bartoníček et al. developed a more complex classification system for the posterior tibial fragment consisting of five types of fragments: extraincisural (type 1), posterolateral extending into the fibular notch (type 2), two-part posteromedial involving the medial malleolus (type 3), large posterolateral triangular (type 4), and irregular (type 5) [9].

More recently, Mason and Molloy introduced a posterior classification system underpinned by a pathomechanistic approach resulting in four types of fracture: small extra-articular posterior malleolar fragments (type 1), fractures of the posterolateral tibia/Volkmann’s area (type 2a), fractures similar to fractures of the posterolateral tibia/Volkmann’s area but with a secondary fragment of the posteromedial tibia (type 2b), and coronal plane fractures of the posterior plafond (type 3) [10]. There are commonalities across these classification systems, with Haraguchi type 1, Mason and Molloy type 2a, and Bartoníček type 2 fractures demonstrating similar morphology and Bartoníček type 3 and Mason and Molloy type 2a fractures demonstrating similar morphology.

For this study, we compared the inter- and intra-rater reliabilities of the Haraguchi et al. [8], Bartoníček et al. [9], and Mason and Molloy [10] classification systems for posterior malleolus fractures. These three systems were chosen for comparison because they represent widely used CT-based approaches to posterior malleolar fracture classification. Reliable classification is critical for consistent surgical decision-making and communication between clinicians.

An earlier version of this article was presented as a poster at the British Orthopaedic Foot and Ankle Society Annual Conference held between March 9 and 11, 2022. We were granted permission to reprint the images.

## Materials and methods

This retrospective diagnostic reliability study reviewed the CT scans of 40 consecutive ankle fractures involving the posterior malleolus that were presented to an institution from May 25 to November 30, 2020. The protocol of the department was that all posterior malleolus fractures undergo CT imaging, in line with the British Orthopaedic Association Standards for Trauma [11]. Ankle fractures that did not have a discrete posterior malleolar component were excluded from the study.

Four assessors reviewed the imaging and classified the fractures according to the Haraguchi, Bartoníček, and Mason and Molloy classification systems. The assessors - two trained foot and ankle consultant surgeon fellows, one foot-and-ankle fellow, and one orthopedic resident - were familiar with these classification systems and had access to the original articles describing the three classification systems. They did not confer on the classifications, nor did they receive any additional training prior to the review period.

The reviews were conducted twice, with one month between them. The assessors were blinded to the identity of the patients and had no previous knowledge of the patients’ management. The collated data were analyzed using R version 4.2.1 software (The R Foundation for Statistical Computing, Vienna, Austria), with the Fleiss kappa statistic being used for inter-observer reliability and the mean Cohen’s kappa for intra-observer reliability. The Landis and Koch interpretation for these kappa values were utilized, with values ranging from 0 to 0.2 classified as slight agreement, values ranging from 0.21 to 0.4 classified as fair agreement, values ranging from 0.41 to 0.6 classified as moderate agreement, values ranging from 0.61 to 0.8 classified as substantial agreement, and values ranging from 0.81 to 1 indicating perfect agreement [12]. In addition, a subgroup analysis was carried out to determine where variation occurred among the assessors.

## Results

The distribution of the classes of fractures in the first round of assessments is shown in Table 1.

**Table 1:** Relative frequencies of fracture classes in the first round of assessments

Classification	Percentage
Bartoníček	
Type 1: Extraincisural fragment	14%
Type 2: Posterolateral fragment	34%
Type 3: Posteromedial, two-part fragment	34%
Type 4: Large, posterolateral triangular fragment	11%
Type 5: Irregular osteoporotic fracture	8%
Haraguchi	
Type 1: Posterolateral-oblique type	43%
Type 2: Medial extension type	41%
Type 3: Small-shell type	17%
Mason and Molloy	
Type 1: Extra-articular	19%
Type 2A: Posterolateral triangle extending into incisura	40%
Type 2B: Secondary posteromedial fragment	36%
Type 3: Coronal plane fracture, whole posterior plafond	6%

**Figure 1:**
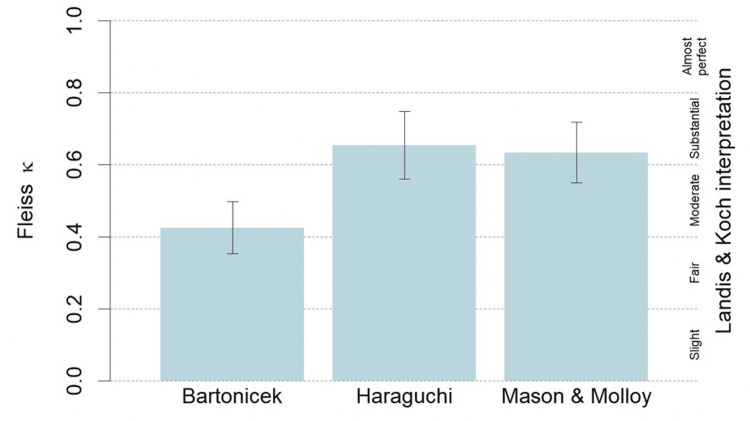
Inter-observer reliability for the Bartoníček, Haraguchi, and Mason and Molloy classification systems (95% confidence intervals).

**Figure 2:**
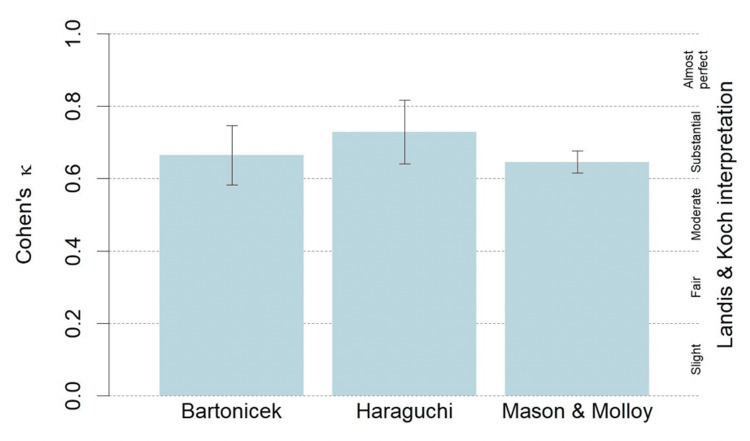
Intra-observer reliability for the Bartoníček, Haraguchi, and Mason and Molloy classification systems (mean±standard deviation).

The inter-observer reliability for the Haraguchi classification and the Mason and Molloy classification was similar, with both demonstrating “substantial” agreement, while the Bartoníček classification demonstrated only “moderate” agreement. All three classifications demonstrated “substantial” agreement with regard to intra-observer reliability. The Haraguchi classification system had the highest inter-observer and intra-observer reliability. The Fleiss kappa and Cohen’s kappa results are detailed in Figures 1, 2.

In total, each posterior malleolus fracture was classified eight times using each classification system, with the four reviewers evaluating each fracture twice. An analysis of each classification system was performed, focusing on the frequency of complete agreement during all eight classification episodes. Again, the Haraguchi classification system demonstrated the highest instance of complete agreement, which occurred 32% of the time for type 1 fractures. In the Mason and Molloy classification, type 2a fractures demonstrated complete agreement 21% of the time, and Bartoníček type 3 fractures did so 17% of the time. 

The frequency of complete agreement across all eight instances of classification was also found to be highest for the Haraguchi classification system and lowest for the Bartoníček classification. Table 2 shows the frequency of agreement across the eight assessments for each classification type within each system.

**Table 2:** Frequency of complete agreement within each classification

Classification	Complete agreement (8/8 assessments)
Haraguchi	
Type 1	32%
Type 2	20%
Type 3	13%
Mason and Molloy	
Type 1	15%
Type 2a	21%
Type 2b	13%
Type 3	12%
Bartoníček	
Type 1	5%
Type 2	12%
Type 3	17%
Type 4	0%
Type 5	0%

## Discussion

An ideal orthopedic classification system enables the clear communication of information, guides treatment, facilitates research to categorize a spectrum of injury, and has prognostic value [13]. Accordingly, agreement among observers and among observations is vital if a classification system is to be reliable. High inter- and intra-observer reliability is essential because consistent classification underpins reproducible surgical decision-making. It has been demonstrated elsewhere that trimalleolar fractures result in worse outcomes than bimalleolar ankle fractures [14]. Accurate assessment and appropriate fixation of posterior malleolus fractures have been shown to improve post-surgery outcomes [2].

As the first classification system developed for posterior malleolar fractures, the Haraguchi classification has been widely used. This descriptive classification is not prognostic, nor does it provide guidance regarding management [8]. This system achieved the highest inter-observer and intra-observer reliability in our study. The reason for this result is uncertain, but the Haraguchi classification categorizes posterior malleolar fractures into only three types, whereas the Mason and Molloy and Bartoníček classification systems categorize the fractures into four or five types, respectively. The greater complexity of these systems may account for the variation in the results that we observed. A causal relationship between the increased complexity of classification systems and worsening inter-observer and intra-observer reliability has been theorized previously [15]. 

The Mason and Molloy classification incorporates the mechanism of injury and suggests surgical approaches and fixation methods [2]. The researchers who devised it have also shown that the morphology of posterior malleolar fractures affects syndesmotic stability and that the use of this classification to guide management improves the outcomes of trimalleolar fractures so that they are similar to the outcomes of bimalleolar ankle fracture fixation [2]. 

The Bartoníček classification system introduced recognition of fibular incisural involvement into the morphology of posterior malleolar fractures [9]. Given the recent evidence of variation in incisural anatomy and the role of the incisura in the bony stability of the syndesmosis, the Bartoníček classification may be important for managing these injuries [16]. In addition, the Bartoníček classification defines when a posterior malleolar fracture should be considered a pilon fracture by excluding fractures in which the entire medial malleolus is a component of the posteromedial fragment [9]. Although the Bartoníček classification introduces concepts that are important for understanding the morphology of posterior malleolar fractures, it demonstrated the lowest inter-observer reliability in the present study, and this result indicates that its potential for use in determining prognosis and management is limited.

There are several commonalities among these classification systems. To begin with, all three systems recognize the most common configuration of the posterolateral Volkmann fragment, which contains the origin of the posterior inferior tibiofibular ligament (Haraguchi type 1, Mason and Molloy type 2A, and Bartoníček type 2). The Bartoníček type 4 fracture incorporates a larger posterolateral fragment, while the Mason and Molloy type 3 fracture involves a large posterior fragment with a vertical shear injury mechanism. Within the Bartoníček classification, the variation in size of the posterolateral fracture between type 2 and type 4 has the potential to reduce reliability. All three classification systems recognize the importance of medial extension of the posterior malleolar fracture, but the Mason and Molloy and Bartoníček classifications describe two fragment components, posterolateral and posteromedial (Mason and Molloy type 2B, Bartoníček type 3). Both the Mason and Molloy and Bartoníček classifications have a category for a large single posterior malleolar fragment that extends medially (Mason and Molloy type 3, Bartoníček type 4). Lastly, all three classification systems have a category for thin posterior malleolar fractures, which are likely indicative of syndesmotic injury (Haraguchi type 3, Mason and Molloy type 1, and Bartoníček type 1). Several factors that may affect outcomes are not incorporated into any of the posterior malleolar classifications. Impacted fragments and joint incongruency have been shown in comparative case series to have a detrimental influence on the rates of post-traumatic arthritis and the outcome scores [5,17].

In a recent study of fractures involving the posterior tibial articular surface, medial impaction fragments appeared to be associated with poorer outcomes compared with fractures that did not display this morphology [18]. These factors could facilitate decisions regarding whether a posterior malleolar fracture is addressed directly and which method of reduction and/or fixation to use and assist in determining the immobilization status and/or duration. Although medial extension of the fracture is considered in all three classification systems, none of them formally recognizes a fracture line involving the tibialis posterior groove, particularly when incarceration of the posterior tibial tendon is observed [19]. Medial extension may be important to consider in the management of posterior malleolar fractures and should be a topic of future research.

This study benefited from the contributions of multiple expert observers with pragmatic knowledge of the classification systems, real-world data, and blinding to previous assessments. Therefore, the findings should be generalizable to comparable clinical settings. We found that the Haraguchi classification system was more reliable than the Bartoníček and Mason and Molloy systems across all domains, though previous research demonstrated high inter-observer reliability [10,20]. Reliability is only one domain in which a classification system must perform in order to have clinical utility. While the Haraguchi system demonstrated the highest reliability, it does not provide prognostic or therapeutic guidance. Conversely, the Mason and Molloy classification, despite its slightly lower reliability, offers clinicians useful therapeutic guidance for management decisions. We were unable to examine the impact of each system on decision-making or surgical planning, techniques, or outcomes.

This study has several limitations. First, it took the form of a retrospective, single-center analysis with a sample size of 40. While adequate for analyzing the reliability of the classification systems, the focus on a single center and use of a relatively small sample size may limit the generalizability of the findings to other institutions or broader patient populations. Second, all of the reviewers were familiar with the three classification systems, but they received no formal training in their use, and this lack of training may have contributed to the variability in their interpretations. In addition, differences in seniority may have influenced interpretation. Lastly, this study focused on radiological classification and did not correlate against treatment decisions, surgical approaches, or patient outcomes.

## Conclusions

We found that the Haraguchi classification system achieved better inter- and intra-observer reliability than the Bartoníček system and the Mason and Molloy system. The inter-observer reliability agreement for the Haraguchi and the Mason and Molloy classifications was substantial. While the Mason and Molloy system showed slightly lower reliability, it offers significant utility for clinicians in decision-making and choosing treatment strategies.

Given the increasing emphasis on the morphology of posterior malleolus fractures, the systems for classifying these fractures must balance reliability, simplicity, and prognostic value. Understanding which classification systems demonstrate higher reliability can help standardise preoperative planning, promote consistency in treatment decisions, and provide a more stable foundation for future studies linking morphology to functional outcomes. Future studies should assess how these systems influence decision-making, treatment strategies, and patient-reported outcome measures.
